# Novel Betaherpesvirus in Bats

**DOI:** 10.3201/eid1606.091567

**Published:** 2010-06

**Authors:** Shumpei Watanabe, Ken Maeda, Kazuo Suzuki, Naoya Ueda, Koichiro Iha, Satoshi Taniguchi, Hiroshi Shimoda, Kentaro Kato, Yasuhiro Yoshikawa, Shigeru Morikawa, Ichiro Kurane, Hiroomi Akashi, Tetsuya Mizutani

**Affiliations:** The University of Tokyo, Tokyo, Japan (S. Watanabe, N. Ueda, K. Iha, S. Taniguchi, K. Kato, Y. Yoshikawa, H. Akashi); Yamaguchi University, Yamaguchi, Japan (K. Maeda, H. Shimoda); Hikiiwa Park Center, Wakayama, Japan (K. Suzuki); National Institute of Infectious Diseases, Tokyo (S. Morikawa, I. Kurane, T. Mizutani); 1 These authors contributed equally to this work.

**Keywords:** RDV, novel betaherpesvirus, bat, viruses, dispatch

## Abstract

Because bats are associated with emerging zoonoses, identification and characterization of novel viruses from bats is needed. Using a modified rapid determination system for viral RNA/DNA sequences, we identified a novel bat betaherpesvirus 2 not detected by herpesvirus consensus PCR. This modified system is useful for detecting unknown viruses.

Since the 1990s, bats have been associated with several emerging zoonotic agents, including Hendra, Nipah, Ebola, lyssa, and severe acute respiratory syndrome coronavirus-like viruses (*1*). Bats seem to have great potential as reservoirs for emerging viruses. Therefore, to understand the role of bats as a host species, identification and characterization of novel viruses from bats is needed. For virus isolation, we have been attempting to establish primary cell cultures from various bats (*2*,*3*). Using a rapid determination system for viral RNA sequences (RDV), we discovered a novel adenovirus and gammaherpesvirus in bats (*2*,*4*). This system, which we simplified to a less laborious one (*5*), is useful for detecting viruses, regardless of virus species (*6*).

## The Study

During June–August, 2008, with the permission of the governor of Wakayama Prefecture, Japan, we caught 8 insectivorous vespertilionid bats, *Miniopterus fuliginosus*, and used their spleens and kidneys to establish primary cell cultures. During passage of the primary spleen adherent cells, cytopathic effect (cell death) was noted at third passage. The collected supernatant was injected into fresh primary kidney cells and caused apparent cytopathic effect at first passage.

Before using the RDV method, we had attempted to detect herpesvirus by nested PCR with the consensus primer sets DFA, ILK, KG1, TGV, and IYG, which were designed according to the consensus-degenerate hybrid oligonucleotide primers program (*7*). These consensus degenerate primers are effective for detecting many herpesviruses from vertebrate hosts. However, in this study they failed to detect any herpesviruses.

We then attempted to detect herpesvirus by using RDV version 3.1, our modification from version 3.0 (*5*). The adapters and primers for construction of the second cDNA library in RDV version 3.1 were newly designed and replaced those used in RDV version 3.0 (Technical Appendix 1). Both adapters have sticky-end structures digested with *Sau*3AI or *Hpy*CH4 IV. RDV version 3.1 can determine an unknown viral cDNA fragment with 64 primer pairs, which we used for constructing the second cDNA library.

With RDV version 3.1, we obtained 4 unknown cDNA fragments, which had no matches in a BLASTn (www.ncbi.nlm.nih.gov/blast/Blast.cgi) search. In a BLASTx search, 1 cDNA fragment (deduced sequence of 29 aa) was homologous to the glycoprotein B (gB) amino acid sequence of the tupaiid herpesvirus 1 (TuHV-1) (79% identity), which belongs to subfamily *Betaherpesvirinae*. We designed new consensus-degenerate hybrid oligonucleotide primers (http://blocks.fhcrc.org/codehop.html) selective for the betaherpesvirus gB and DNA polymerase (DPOL) genes, and we determined the complete gB sequence and the partial DPOL sequence of the isolated virus (5,029 bp, DNA Data Bank of Japan accession no. AB517983). BLAST search indicated that the complete gB sequence was novel and most similar to that of TuHV-1 (59% aa sequence identity) (Appendix Figure). We named the isolated virus bat betaherpesvirus 2 (BatBHV-2).

We constructed a phylogenetic tree by using the neighbor-joining method with the gB amino acid sequence and the available sequences of known herpesviruses (Figure). The phylogenetic tree based on betaherpesvirus gB genes showed that BatBHV-2 is most closely related to TuHV-1 and caviid herpesvirus 2 (guinea pig cytomegalovirus). The subfamily *Betaherpesvirinae* consists of the genera *Cytomegalovirus*, *Muromegalovirus,* and *Roseoloviru*s. TuHV-2 and caviid herpesvirus 2 are species unassigned to any genus in the subfamily *Betaherpesvirinae*.

**Figure:**
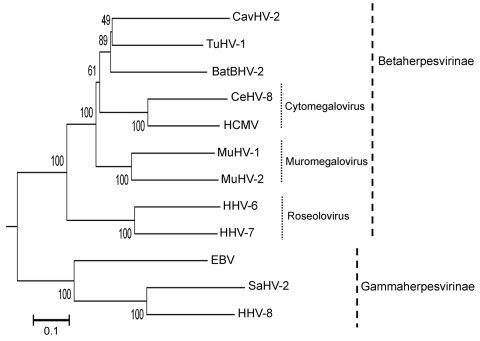
Phylogenetic tree based on the deduced amino acid sequences of complete glycoprotein B. The percentage of replicate trees in which the associated taxa clustered together in the bootstrap test (1,000 replicates) is shown next to the branches. The tree is drawn to scale, with branch lengths in the same units as those of the evolutionary distances used to infer the phylogenetic tree. The tree was rooted to herpes simplex virus type 1 (X14112). The evolutionary distances were computed by using the Poisson correction method and are in units of the number of amino acid substitutions per site. All positions containing gaps and missing data were eliminated from the dataset. The final dataset included a total of 698 positions. Phylogenetic analyses were conducted in MEGA4 (*8*). The herpesviruses used for comparison and their accession numbers are as follows: Epstein-Barr virus 1 (EBV, NC_007605), caviid herpesvirus (CavHV-2, FJ355434); mouse cytomegalovirus (MuHV-1, NC_004065), human cytomegalovirus (HCMV, X17403), human herpesvirus 6 (HHV-6, AF157706), human herpesvirus 7 (HHV-7, AF037218), human herpesvirus 8 (HHV-8, AF148805), rat cytomegalovirus (MuHV-2, NC_002512), cercopithecine herpesvirus 8 (CeHV-8, AY186194), saimiriine herpesvirus 2 (SaHV-2, NC_001350), and tupaiid herpesvirus 1 (TuHV-1, AF281817). Scale bar indicates evolutionary distance.

In May 2009, we collected, again with permission, another 50 bats belonging to 1 species, *M. fuliginosus,* from the same location for an epizootologic study (Technical Appendix 2). Spleens and blood were collected from all bats, and other organs (liver, kidney, lung, brain, intestine, trachea, and urinary bladder) were collected from 10 bats. Nested PCR was performed by using specific primers selective for the DPOL gene of BatBHV-2, and PCR products were subjected to direct sequencing. Viral nucleotide sequences were obtained from 4 of the 50 spleen samples. Each nucleotide sequence showed complete identity to the partial DPOL sequence of the BatBHV-2. Other organs and serum collected from 2 of the bats were also tested by nested PCR, and viral DNA was detected in the liver, kidneys, and lungs of both bats.

## Conclusions

Although PCRs with consensus primers effectively detect known and unknown viruses, they failed to detect BatBHV-2, possibly because of minor mismatches between the sequences of BatBHV-2 and the primer sets (TGV, IYG, and KG1). The variety of virus sequences and gene mutations often prevents successful amplification of virus genes. RDV, however, can detect viral cDNA fragments independent of virus species and thus is useful as a first-choice tool for identifying emerging known and unknown viruses in animals and humans.

BLAST search showed that the complete gB sequence of the isolated virus was novel and most similar to that of TuHV-1. Recently, bats have been described as hosts for herpesviruses in several countries in Europe, America, Africa, and Asia (*4**,**9**,**10*). Wibbelt et al. reported that the partial DPOL sequence (175 bp) of a betaherpesvirus, bat betaherpesvirus 1 (BatBHV-1), was obtained from several insectivorous bat species (*10*). Although the length of the BatBHV-1 sequence was short, similarity between BatBHV-1 and BatBHV-2 was relatively high (58%). BatBHV-1 is most similar to TuHV-1(61%). These findings suggest that BatBHV-2 is a different species than BatBHV-1.

Our epizootologic study found relatively high (8%) prevalence of BatBHV-2 in insectivorous bats. Although the virus genome was detected in a few parenchymal organs by nested PCR, no amplification was possible for serum, intestine, or urinary bladder samples, which may exclude apparent virus shedding by the bats. In addition, all 50 bats collected appeared clinically healthy. To understand the life cycle of this virus, the possibility of a latent infection in these insectivorous bats must be explored.

## Supplementary Material

Appendix Figure Phylogenetic tree based on the deduced amino acid sequences of complete glycoprotein B. The percentage of replicate trees in which the associated taxa clustered together in the bootstrap test (1,000 replicates) is shown next to the branches. The tree is drawn to scale, with branch lengths in the same units as those of the evolutionary distances used to infer the phylogenetic tree. The tree was rooted to herpes simplex virus type 1 (X14112). The evolutionary distances were computed by using the Poisson correction method and are in units of the number of amino acid substitutions per site. All positions containing gaps and missing data were eliminated from the dataset. The final dataset included a total of 698 positions. Phylogenetic analyses were conducted in MEGA4 (*8*). The herpesviruses used for comparison and their accession numbers are as follows: Epstein-Barr virus 1 (EBV, NC_007605), caviid herpesvirus (CavHV-2, FJ355434); mouse cytomegalovirus (MuHV-1, NC_004065), human cytomegalovirus (HCMV, X17403), human herpesvirus 6 (HHV-6, AF157706), human herpesvirus 7 (HHV-7, AF037218), human herpesvirus 8 (HHV-8, AF148805), rat cytomegalovirus (MuHV-2, NC_002512), cercopithecine herpesvirus 8 (CeHV-8, AY186194), saimiriine herpesvirus 2 (SaHV-2, NC_001350), and tupaiid herpesvirus 1 (TuHV-1, AF281817). Scale bar indicates evolutionary distance.

Technical Appendix 1 System for Rapid Determination of Viral RNA Sequences, Version 3.1.

Technical Appendix 2 Epizootologic Study.
