# Supplementary figures and images for: Novel Betaherpesvirus in Bats
Source: Emerg Infect Dis. 2010 Jun;16(6):986–8. doi: 10.3201/eid1606.091567 (PMC3086227; doi:10.3201/eid1606.091567)

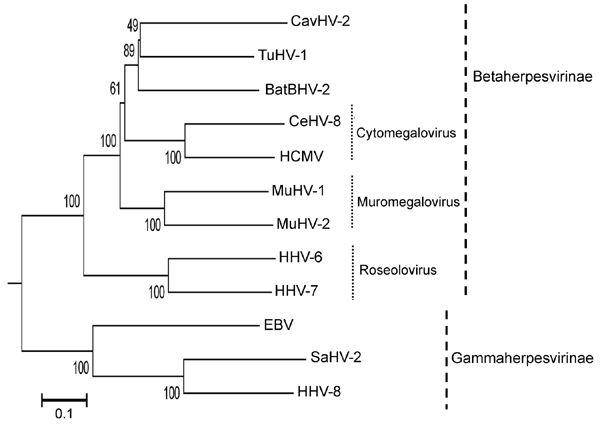

Supplement: Appendix Figure — Phylogenetic tree based on the deduced amino acid sequences of complete glycoprotein B. The percentage of replicate trees in which the associated taxa clustered together in the bootstrap test (1,000 replicates) is shown next to the branches. The tree is drawn to scale, with branch lengths in the same units as those of the evolutionary distances used to infer the phylogenetic tree. The tree was rooted to herpes simplex virus type 1 (X14112). The evolutionary distances were computed by using the Poisson correction method and are in units of the number of amino acid substitutions per site. All positions containing gaps and missing data were eliminated from the dataset. The final dataset included a total of 698 positions. Phylogenetic analyses were conducted in MEGA4 (8). The herpesviruses used for comparison and their accession numbers are as follows: Epstein-Barr virus 1 (EBV, NC_007605), caviid herpesvirus (CavHV-2, FJ355434); mouse cytomegalovirus (MuHV-1, NC_004065), human cytomegalovirus (HCMV, X17403), human herpesvirus 6 (HHV-6, AF157706), human herpesvirus 7 (HHV-7, AF037218), human herpesvirus 8 (HHV-8, AF148805), rat cytomegalovirus (MuHV-2, NC_002512), cercopithecine herpesvirus 8 (CeHV-8, AY186194), saimiriine herpesvirus 2 (SaHV-2, NC_001350), and tupaiid herpesvirus 1 (TuHV-1, AF281817). Scale bar indicates evolutionary distance. [file 09-1567-appF-s1.gif]
