# Supplementary material for: Novel Betaherpesvirus in Bats
Source: Emerg Infect Dis. 2010 Jun;16(6):986–8. doi: 10.3201/eid1606.091567 (PMC3086227; doi:10.3201/eid1606.091567)
Supplement: Technical Appendix 1 — System for Rapid Determination of Viral RNA Sequences, Version 3.1. [file 09-1567-Techapp1-s2.pdf]

DOI: 10.3201/eid1606.091567

Novel Betaherpesvirus in Bats

Technical Appendix 1

System for Rapid Determination of Viral RNA Sequences, Version 3.1

The system for rapid determination of viral RNA sequences (RDV) version 3.0 (*1*) was modified to simplify the procedure. The modified method was designated version 3.1. Adapters and primers for construction of second cDNA library in RDV version 3.1 were newly designed and used instead of those in RDV version 3.0 (see Figure below. A: adaptors, B: primers). Both adapters have sticky-end structures digested with *Sau*3AI or *Hpy*CH4IV. The RDV version 3.1 method includes the 4 procedures described below.

1. RNA extraction. To eliminate contaminating cellular RNA and DNA from the samples, culture supernatant was treated with DNase I and RNase A as described previously (*2*), following RNA extraction by using a Total RNA isolation mini kit (Agilent Technologies, Santa Clara, CA, USA).
2. Construction of first cDNA library. A whole-transcriptome amplification system (WTA; Sigma-Aldrich, Saint Louis, MO, USA) was used to amplify double-stranded viral cDNA as described previously (*3*).
3. Construction of second cDNA library. After the first cDNA library purification with the MonoFas DNA isolation system (GL Sciences, Tokyo, Japan), DNA was

digested with 20 U of *Hpy*CH4IV (New England Biolabs, Ipswich, MA, USA) and *Sau*3AI (New England Biolabs) at 37°C for 60 min, and the digested DNA was again purified by using MonoFas. For construction of the second cDNA library, aliquots of 2.5 uL of DNA solution, 2.5 uL of distilled water, 2.5 uL of RDV-Adaptor-*Sau*3AI adaptor (10 uM) and RDV-Adaptor-*Hpy*CH4IV adaptor (10 uM) were mixed. The sequences and structures of the adapters are shown in figure below. A ligation-convenience kit (Nippon Gene, Toyama, Japan) was used for adaptor ligation. The DNA solution and 10 uL of ligation mix were reacted at 16°C for 30 min, and the DNA was isolated by using MonoFas. The second cDNA library was amplified by PCR with specially designed primer sets; the forward primers had 5 nt including GATC (*Sau*3AI-digested sequence) and 1 variable nucleotide added to the 3′ end of the RDV-Adaptor-*Sau*3AI sequence, and the reverse primers had 6 nt including ACGT (*Hpy*CH4IV-digested sequence) and 2 variable nucleotides added to the 3′ end of the RDV-Adaptor-*Hpy*CH4IV sequence. The PCR mixture was prepared by mixing 15 uL of AmpliTaq Gold PCR Master Mix containing AmpliTaq Gold, 0.5 uL of forward primer for RDV-Adaptor-*Sau*3AI (10 uM), 0.5 uL of reverse primer for RDV-Adaptor-*Hpy*CH4IV (10 uM), 1 uL of DNA solution, and 13 uL of distilled water. PCR was performed by using 64 primers under the conditions described previously (*1*).

4. Direct sequencing. After electrophoresis of PCR products on agarose gels, bands >120 bp were excised, and DNA was extracted from the gel by using MonoFas. Direct sequencing was performed by using the forward or reverse primer.

References

1. Omatsu T, Watanabe S, Akashi H, Yoshikawa Y. Biological characters of bats in relation to natural reservoir of emerging viruses. Comp Immunol Microbiol Infect Dis. 2007;30:357–74. PubMed DOI: 10.1016/j.cimid.2007.05.006

2. Watanabe S, Ueda N, Iha K, Masangkay JS, Fujii H, Alviola P, et al. Detection of a new bat gammaherpesvirus in the Philippines. Virus Genes. 2009;39:90–3. PubMed DOI: 10.1007/s11262-009-0368-8

3. Sakai K, Mizutani T, Fukushi S, Saijo M, Endoh D, Kurane I, et al. An improved procedure for rapid determination of viral RNA sequences of avian RNA viruses. Arch Virol. 2007;52:1763–5. DOI: 10.1007/s00705-007-0999-9

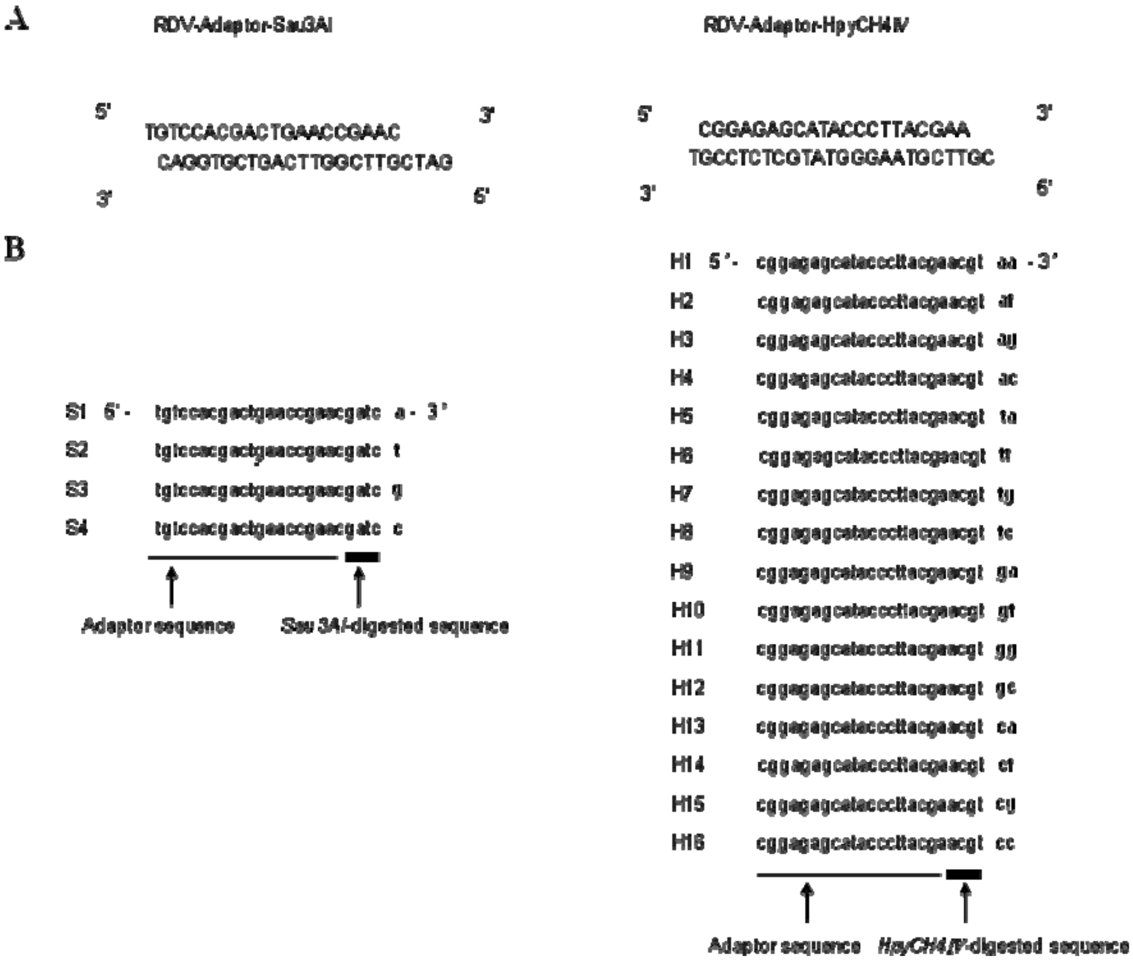
A
RDV-Adaptor-Sau3AI
5' TGTCCACGACTGAACCGAAC 3'
3' CAGGTGCTGACTTGGCTTGCTAG 5'
RDV-Adaptor-HpyCH4IV
5' CGGAGAGCATACCCTTACGAA 3'
3' TGCCTCTCGTATGGGAATGCTTGC 5'
B
S1 5'- tgtccacgactgaaccgaacgatc a - 3'
S2 tgtccacgactgaaccgaacgatc t
S3 tgtccacgactgaaccgaacgatc g
S4 tgtccacgactgaaccgaacgatc c
Adaptor sequence
Sau 3AI-digested sequence
H1 5'- cggagagcatacccttacgaacgt aa - 3'
H2 cggagagcatacccttacgaacgt at
H3 cggagagcatacccttacgaacgt ag
H4 cggagagcatacccttacgaacgt ac
H5 cggagagcatacccttacgaacgt ta
H6 cggagagcatacccttacgaacgt tt
H7 cggagagcatacccttacgaacgt tg
H8 cggagagcatacccttacgaacgt tc
H9 cggagagcatacccttacgaacgt ga
H10 cggagagcatacccttacgaacgt gt
H11 cggagagcatacccttacgaacgt gg
H12 cggagagcatacccttacgaacgt gc
H13 cggagagcatacccttacgaacgt ca
H14 cggagagcatacccttacgaacgt ct
H15 cggagagcatacccttacgaacgt cg
H16 cggagagcatacccttacgaacgt cc
Adaptor sequence
HpyCH4IV-digested sequence
