# Supplementary material for: Novel Betaherpesvirus in Bats
Source: Emerg Infect Dis. 2010 Jun;16(6):986–8. doi: 10.3201/eid1606.091567 (PMC3086227; doi:10.3201/eid1606.091567)
Supplement: Technical Appendix 2 — Epizootologic Study. [file 09-1567-Techapp2-s3.pdf]

DOI: 10.3201/eid1606.091567

Novel Betaherpesvirus in Bats

Technical Appendix 2

Epizootologic Study

Fifty bats (nos. Mf01–Mf50) belonging to 1 species, *M. fuliginosus*, were collected; 28 were female, 25 of which were pregnant. Spleens and serum samples were collected from all bats, and other organs (liver, kidney, lung, brain, intestine, trachea, and urinary bladder) were collected from 10 bats (nos. Mf1–Mf10). DNA was extracted from each spleen sample and subjected to viral genome detection. Nested PCR was performed by using specific primers targeting the DNA polymerase (DPOL) gene of bat betaherpesvirus 2 (BatBHV-2), and PCR products were subjected to direct sequencing. Viral nucleotide sequences were obtained from 4 (bats. Mf4, Mf7, Mf14, Mf33) of the 50 spleen samples. Bats Mf4, Mf7 and Mf33 were pregnant. The obtained nucleotide sequences showed complete identity to the partial DPOL sequence of the isolated virus. Other organs and serum collected from bats Mf4 and Mf7 were also tested by nested PCR. Viral DNA was detected in organs (liver, kidney, and lung) of each of these bats. The pattern of viral distribution in organs was identical between bats Mf4 and Mf7. These results were summarized below.

Results of nested PCR

Bat no.	Sex	Spleen	Intestine	Liver	Kidney	Bladder	Lung	Trachea	Brain	Sera
Mf4	F	+	-	+	+	-	+	-	-	-
Mf7	F	+	-	+	+	-	+	-	-	-
Mf14	F	+	ND	ND	ND	ND	ND	ND	ND	ND
Mf33	M	+	ND	ND	ND	ND	ND	ND	ND	ND

ND Not done
